# Supplementary material for: Developing a COVID-19 mortality risk prediction model when individual-level data are not available
Source: Nat Commun. 2020 Sep 7;11:4439. doi: 10.1038/s41467-020-18297-9 (PMC7477233; doi:10.1038/s41467-020-18297-9)
Supplement: Supplementary file 1 — Supplementary Information [file 41467_2020_18297_MOESM1_ESM.pdf]

Supplementary Material

Developing a COVID-19 mortality risk prediction model when individual level data is not available

Noam Barda[1,2,3], Dan Riesel[1], Amichay Akriv[1], Joseph Levy[1], Uriah Finkel[1], Gal Yona[4], Daniel Greenfeld[5], Shimon Sheiba[5], Jonathan Somer[5], Eitan Bachmat[3], Guy N. Rothblum[4], Uri Shalit[5], Doron Netzer[6], Ran Balicer[1,2], Noa Dagan[1,2,3]

Contents

Supplementary Table 1: Population table for baseline model 2

Supplementary Table 2: Corrections table for the "multi-calibration" recalibration procedure 4

Supplementary Table 3: Sensitivity analysis results for the discrimination performance considering the composite outcome (severe cases and mortality) 5

Supplementary Table 4: Variable and outcome definitions 6

Supplementary Figure 1: Odds ratio as a function of variable values for the baseline predictor 8

Supplementary Figure 2: Cumulative Distribution Function of time-to-death among CHS COVID-19 patients 9

Supplementary Methods 1: Transformation of SHAP values to odds ratios 10

Supplementary Methods 2: Pseudo-code of the "multi-calibration" algorithm 11

Supplementary Methods 3: Technical description of adjustment procedure 12

References 14

Supplementary Table 1: Population table for baseline model

Variable	Train/Validation	Test	Missing (%)
**Overall**	735,000	315,000	
**Outcome**, n (%)			0.0
No	726,749 (98.9)	311,533 (98.9)	
Yes	8,251 (1.1)	3,467 (1.1)	
**Age**, Mean (SD)	41.00 (21.26)	40.99 (21.23)	0.0
**Sex**, n (%)			0.0
Female	378,617 (51.5)	162,144 (51.5)	
Male	356,383 (48.5)	152,856 (48.5)	
**Pack years**, Mean (SD)	23.65 (24.07)	23.52 (23.31)	75.5
**Chronic Respiratory Disease**, n (%)			0.0
No	669,347 (91.1)	286,961 (91.1)	
Yes	65,653 (8.9)	28,039 (8.9)	
**Cardiovascular Disease**, n (%)			0.0
No	657,985 (89.5)	281,953 (89.5)	
Yes	77,015 (10.5)	33,047 (10.5)	
**Diabetes**, n (%)			0.0
No	655,383 (89.2)	280,804 (89.1)	
Yes	79,617 (10.8)	34,196 (10.9)	
**Malignancy**, n (%)			0.0
No	694,706 (94.5)	297,718 (94.5)	
Yes	40,294 (5.5)	17,282 (5.5)	
**Hypertension**, n (%)			0.0
No	612,797 (83.4)	262,802 (83.4)	
Yes	122,203 (16.6)	52,198 (16.6)	
**COPD**, n (%)			0.0
No	721252 (98.1)	309082 (98.1)	
Yes	13748 (1.9)	5918 (1.9)	
**Wheezing/Dyspnea diagnosis**, Mean (SD)	0.05 (0.47)	0.05 (0.49)	0.0
**Albumin**, Mean (SD)	4.24 (0.38)	4.24 (0.38)	64.1
**Red cell distribution width**, Mean (SD)	13.69 (1.32)	13.69 (1.31)	49.9
**C-Reactive Peptide**, Mean (SD)	1.16 (2.80)	1.14 (2.73)	86.6
**Urea**, Mean (SD)	31.49 (14.34)	31.38 (14.37)	50.4
**Lymphocyte%**, Mean (SD)	31.12 (9.07)	31.16 (9.09)	43.7
**Chloride**, Mean (SD)	103.94 (3.45)	103.95 (3.46)	94.8

**Creatinine**, Mean (SD)	0.79 (0.41)	0.79 (0.41)	46.6
**High Density Lipoprotein**, Mean (SD)	49.08 (13.07)	49.04 (13.04)	51.7
**Duration of hospitalizations**, Mean (SD)	0.55 (5.72)	0.54 (5.86)	0.0
**Count of hospitalizations**, Mean (SD)	0.10 (0.45)	0.10 (0.45)	0.0
**Count of ambulance rides**, Mean (SD)	0.03 (0.26)	0.03 (0.27)	0.0
**Count of Sulfonamide dispenses**, Mean (SD)	0.15 (1.22)	0.15 (1.21)	0.0
**Count of Anti-cholinergic dispenses**, Mean (SD)	0.09 (0.91)	0.09 (0.91)	0.0
**Count of Glucocorticoid dispenses**, Mean (SD)	0.16 (0.94)	0.16 (0.93)	0.0

Population characteristics for the three population sets used to build and validate the baseline model.

Abbreviations: SD, Standard Deviation;

Supplementary Table 2: Corrections table for the "multi-calibration" recalibration procedure

**Total Corrections**	23	
	**Sex**	
**Age Group**	**Male**	**Female**
10-19	+0.003	-0.011214
20-29	+0.00368	-0.010539
30-39	+0.00086	-0.013356
40-49	+0.00723	-0.006987
50-59	+0.01217	-0.002049
60-69	+0.02944	+0.01523
70-79	+0.05912	+0.0449
80+	+0.08484	+0.07063

Total number of corrections performed and cumulative magnitude of the correction for each subgroup of age and sex.

Supplementary Table 3: Sensitivity analysis results for the discrimination performance considering the composite outcome (severe cases and mortality)

Model	Metric	Value
COVID-19 Model	AUROC	0.901, 95% CI: 0.881-0.920
COVID-10 Model 10% risk cut-off	Percent Positive	8%, 95% CI: 7%-9%
	Sensitivity	53%, 95% CI: 47%-60%
	PPV	37%, 95% CI: 32%-43%
COVID-10 Model 5% risk cut-off	Percent Positive	15%, 95% CI: 14-16%
	Sensitivity	73%, 95% CI: 67%-79%
	PPV	27%, 95% CI: 24%-31%

Abbreviations: AUROC, Area Under the Receiver Operating Characteristic curve; PPV, Positive Predictive Value

Supplementary Table 4: Variable and outcome definitions

Variable Definitions	Category	Units	Time Frame (prior to index date)	Details	Mechanism of feature selection	Potential for missing values
Outcome	Diagnoses	No / Yes	NA	ICD9 codes 48[0-8]*, 46[0-6]*, 490*, 510.9*, 511.0, 511.89, 518.82, 785.52 or positive influenza PCR	NA	Always available (cannot be missing)
Age	Demographics	Years	Current	Age in full years	From the top-features list	Always available (cannot be missing)
Sex	Demographics	Male / Female	Current		Added manually	Always available (cannot be missing)
Pack years	Clinical Covariates	Count	Last recorded value in last 1 year	#years smoked X average number of packs per year	From the top-features list	Cannot be missing (if nonsmoker then 0)
COPD	Diagnoses	No / Yes	Ever	As per Clalit's chronic disease registry	From the top-features list	Cannot be missing (if no documentation is available then the variable is set to “No”)
Number of wheezing / dyspnea diagnoses	Diagnoses	Count	Cumulative count over 1 year	ICD9: 786.0	From the top-features list	Cannot be missing (if there is no documentation of a relevant diagnosis the variable is set to 0)
Albumin	Labs	g/dl	Last recorded value in last 1 year		From the top-features list	Missing if the lab test was not done in the last year.
Red cell distribution width	Labs	%	Last recorded value in last 1 year		From the top-features list	Missing if the lab test was not done in the last year.
C-Reactive Peptide	Labs	mcg/ml	Last recorded value in last 1 year		From the top-features list	Missing if the lab test was not done in the last year.
Urea	Labs	mg/dl	Last recorded value in last 1 year		From the top-features list	Missing if the lab test was not done in the last year.
Lymphocyte	Labs	%	Last recorded value in last 1 year		From the top-features list	Missing if the lab test was not done in the last year.
Chloride	Labs	mEq/L	Last recorded value in last 1 year		From the top-features list	Missing if the lab test was not done in the last year.
Creatinine	Labs	mg/dl	Last recorded value in last 1 year		From the top-features list	Missing if the lab test was not done in the last year.
High Density Lipoprotein	Labs	mg/dl	Last recorded value in last 1 year		From the top-features list	Missing if the lab test was not done in the last year.

Duration of hospitalizations	Healthcare Utilization	Days	Cumulative count over 1 year		From the top-features list	Cannot be missing (if there were no hospitalizations then the variable is set to 0)
Count of hospitalizations	Healthcare Utilization	Count	Cumulative count over 1 year		From the top-features list	Cannot be missing (if there were no hospitalizations then the variable is set to 0)
Count of ambulance rides	Healthcare Utilization	Count	Cumulative count over 1 year		From the top-features list	Cannot be missing (if there were no ambulance rides then the variable is set to 0)
Count of Sulfonamide dispenses	Drugs	Count	Cumulative count over 1 year	ATC4: C03CA	From the top-features list	Cannot be missing (if there were no drug dispenses then the variable is set to 0)
Count of Anti-cholinergic dispenses	Drugs	Count	Cumulative count over 1 year	ATC4: R03BB	From the top-features list	Cannot be missing (if there were no drug dispenses then the variable is set to 0)
Count of Glucocorticoid dispenses	Drugs	Count	Cumulative count over 1 year	ATC4: H02AB	From the top-features list	Cannot be missing (if there were no drug dispenses then the variable is set to 0)
Chronic Respiratory Disease	Diagnoses	No / Yes	Ever	As per Clalit's chronic disease registry	Added manually	Cannot be missing (if no documentation is available then the variable is set to “No”)
Cardiovascular Disease	Diagnoses	No / Yes	Ever	As per Clalit's chronic disease registry	Added manually	Cannot be missing (if no documentation is available then the variable is set to “No”)
Diabetes	Diagnoses	No / Yes	Ever	As per Clalit's chronic disease registry	Added manually	Cannot be missing (if no documentation is available then the variable is set to “No”)
Malignancy	Diagnoses	No / Yes	Ever	As per Clalit's chronic disease registry	Added manually	Cannot be missing (if no documentation is available then the variable is set to “No”)
Hypertension	Diagnoses	No / Yes	Ever	As per Clalit's chronic disease registry	Added manually	Cannot be missing (if no documentation is available then the variable is set to “No”)

Caption: A list of the variables used in the model, including their type, units, time frame of extraction, definitions, how they were selected and potential for missingness.

All variables were extracted at or before February 1st, 2020, prior to the onset of the COVID-19 pandemic in Israel.

Abbreviations: COPD, Chronic Obstructive Pulmonary Disease; g, gram; mcg, microgram; mEq, mili-equivalent; ml, milliliter; dl, deciliter; L, liter; PCR, Polymerase Chain Reaction; ICD9, International Classification of Disease, 9th revision; ATC4, Anatomical Therapeutic Chemical (ATC) Classification System, 4th level;

Supplementary Figure 1: Odds ratio as a function of variable values for the baseline predictor

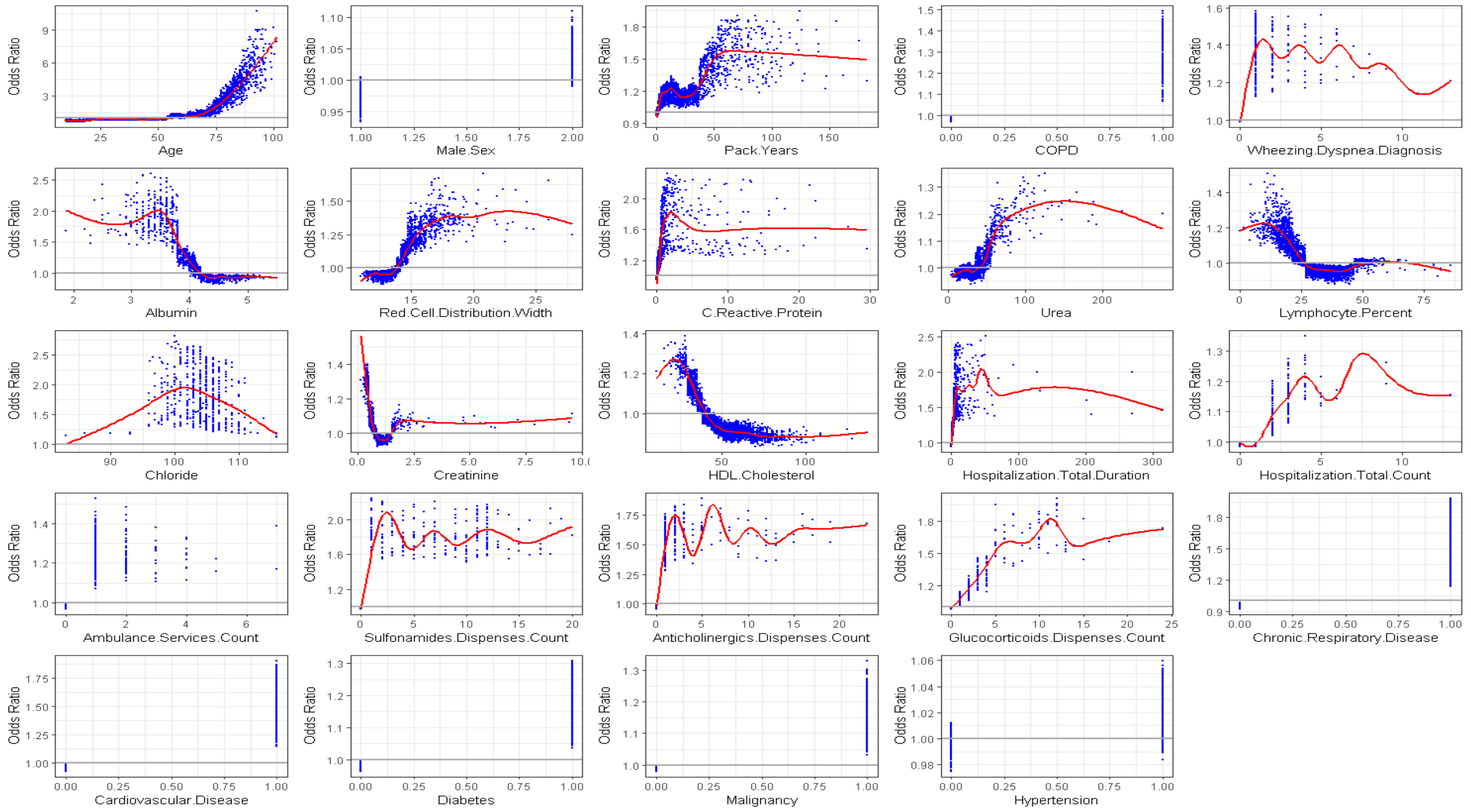

A plot of the odds ratios for different values of the predictors in the baseline model, using SHAP values[1]. A smoothed red line is fit to the curves and a horizontal grey line is drawn at odds ratio = 1.

All figures use a random sample of patients from this same population, n=10,000 unique patients.

Abbreviations: SHAP, SHapley Additive exPlanations; COPD, Chronic Obstructive Pulmonary Disease; HDL, High Density Lipoprotein;

Supplementary Figure 2: Cumulative Distribution Function of time-to-death among CHS COVID-19 patients

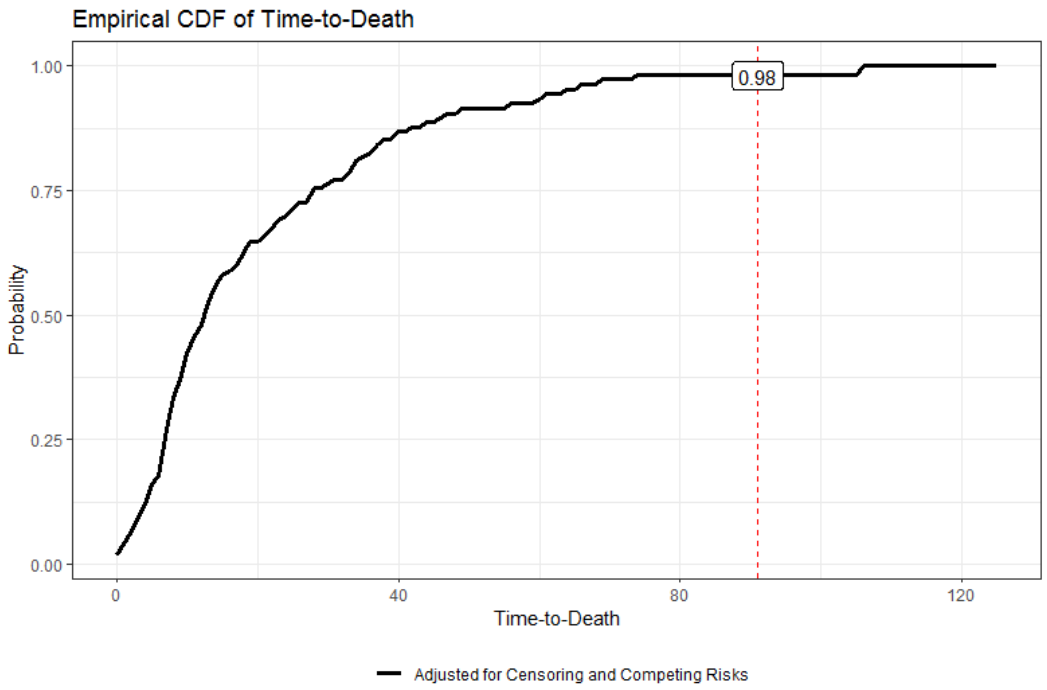

Empirical cumulative distribution function for time-to-death of all COVID-19 patients in CHS' database. The black line shows the cumulative incidence as calculated after accounting for censoring and the "competing risk" of cure, derived using the Aalen-Johansen estimator[2]. The vertical dashed line denotes the follow-up time used in the study, 91 days.

The figure uses all COVID-19 patients among the Clalit Health Services insured population until the extraction date (July 16th, 2020), n=16,049 unique patients.

Abbreviations: CDF, Cumulative Distribution Function; CHS, Clalit Health Services; COVID-19, Corona Virus Disease 2019;

Supplementary Methods 1: Transformation of SHAP values to odds ratios

In Shapley analysis for binary outcomes, the log-odds of the outcome probability is mathematically expressed as a summation of the SHAP values of each of the covariates. The exponent of a single SHAP value (similar to logistic regression), is thus the ratio of the odds of the outcome given that SHAP value divided by the baseline odds of the outcome (using only the intercept), i.e. the odds ratio.

For example, in a model with a single predictor, with x_i marking the SHAP value of that predictor:

- $\begin{cases} \log\left(\frac{p_0}{1-p_0}\right) = x_0 \\ \log\left(\frac{p_1}{1-p_1}\right) = x_0 + x_1 \end{cases}$ =>
- $\log\left(\frac{\frac{p_1}{1-p_1}}{\frac{p_0}{1-p_0}}\right) = \log\left(\frac{p_1}{1-p_1}\right) - \log\left(\frac{p_0}{1-p_0}\right) = x_0 + x_1 - x_0 = x_1$ =>
- $\frac{\frac{p_1}{1-p_1}}{\frac{p_0}{1-p_0}} = \mathrm{e}^{x_1}$

Supplementary Methods 2: Pseudo-code of the "multi-calibration" algorithm

1. **Input**: An initial predictor $\boldsymbol{p}'$, a collection of subpopulations $\boldsymbol{C}$, a training set $\boldsymbol{D} = \{(x_i, y_i)_{i=1}^{m}\}$ and a violation parameters $\alpha > 0$
2. **Output:** a post-processed predictor $\boldsymbol{p}$ satisfying $(\boldsymbol{C}, \alpha)$-multiaccuracy on $\boldsymbol{D}$
3. $\boldsymbol{p} \leftarrow \boldsymbol{p}'$
4. $done \leftarrow False$
5. **while** $\neg done$ **do**
    - 5.1. $done \leftarrow True$
    - 5.2. **foreach** $\boldsymbol{S} \in \boldsymbol{C}$ **do** // iterate over subpopulations
        - 5.2.1. $\Delta_{\boldsymbol{S}} = \frac{\mathbf{1}}{|\boldsymbol{S} \cap \boldsymbol{D}|}\left(\sum_{x_i \in \boldsymbol{S}} y_i - \sum_{x_i \in \boldsymbol{S}} p_i\right)$ // magnitude of violation on $\boldsymbol{S}$
        - 5.2.2. **If** $|\Delta_S| > \alpha$ **then**
            - 5.2.2.1.1. $\boldsymbol{p} \leftarrow \boldsymbol{p} + \Delta_{\boldsymbol{S}} \mathbf{1}_S$ // update predictor by "nudging" $\boldsymbol{S}$
            - 5.2.2.1.2. $done \leftarrow False$
        - 5.2.3. **end if**
    - 5.3. **end foreach**
6. **end while**
7. **return p**

Detailed pseudo-code of the multi-calibration algorithm, as described in Hebert-Johnson et al.[3]
In this study, the algorithm accepts as input the predictions ($\boldsymbol{p}'$) from the baseline model and the collection of subpopulations ($\boldsymbol{C}$) defined by intersections of age and sex groups. It returns predictions recalibrated to COVID-19 outcome rates, which replace the (usually empirically derived) outcomes y_i.

Supplementary Methods 3: Technical description of adjustment procedure

Purpose: to adjust predictions to external one-way conditional probabilities.

Inputs:

- Baseline predictions for another outcome on the entire population of interest, $P(Y_{old}|\boldsymbol{X})$, conditioned on as many independent variables as wanted.
- Published one-way conditional probabilities for the outcome of interest, from an external population, conditional on n variables. $P_{external}(Y_{new}|X_i), I = 1..n$
- A co-occurrence matrix $\boldsymbol{M}_{n\times n}$, where $\boldsymbol{M}_{i,j} = P_{external}(X_j|X_i)$ in the population on which the one-way conditional probabilities were published
- Rates of the different population characteristics in the local population, $P_{local}(X_i), i = 1..n$

Process:

- By solving a system of linear equations, $P_{external}(Y|.) = Mw$, the n coefficients for each variable (W_i) are calculated.
- These coefficients are used to derive $P_{local}(Y|.)$ by multiplying them with the local population characteristics.

Steps:

1. <u>Solving for the coefficients of the linear probability model</u>
   This is done by deriving the coefficients for each independent variable in a linear probability model.
   That is, the model is $P(Y) = W_1P(X_1) + \cdots + W_nP(X_n)$, and the coefficients are solved for by solving a system of $i = 1..n$ equations:
   $P_{external}(Y|X_i) = W_iP_{external}(X_1|X_i) + \cdots + W_nP_{external}(X_n|X_i)$

   Where $P(X_i|X_j)$ is the i,j entry of the $\boldsymbol{M}$ matrix.

2. <u>Adjust external one-way conditionals to the local population</u>

With the coefficients in hand, rates for the local population are calculated using the local probabilities of the independent variables, for $i = 1..n$
$P_{local}(Y|X_i) = W_i P_{local}(X_1|X_i) + \cdots + W_n P_{local}(X_n|X_i)$

3. Recalibrate the predictions for the baseline outcome to the outcome rates of the new outcome
   With the calculated local outcome rates in hand, the baseline predictions are adjusted using the multi-accuracy algorithm depicted in Supplementary Methods 2.

When the process terminates, we have $P(Y_{new}|\boldsymbol{X})$ for the entire population of interest.

References

1. Lundberg, S.M. & Lee, S.-I. A unified approach to interpreting model predictions. in *Advances in neural information processing systems* 4765-4774 (2017).
2. Borgan, Ø. A alen–J ohansen Estimator. *Wiley StatsRef: Statistics Reference Online*, 1-13 (2014).
3. Hébert-Johnson, U., Kim, M.P., Reingold, O. & Rothblum, G.N. Calibration for the (computationally-identifiable) masses. *arXiv preprint arXiv:1711.08513* (2017).
